# Helicobacter pylori Infection and Irritable Bowel Syndrome: A Systematic Review and Meta-Analysis With a Narrative Review of the Evidence for Inflammatory Bowel Disease

**DOI:** 10.7759/cureus.107022

**Published:** 2026-04-14

**Authors:** Hanan Raed Mushtaha Ahmad, Zaina Rawashdeh, Ahmed Rawshdeh, Yazan A Kawafha, Osama M Aldeeb, Aseel A Alawadi, Nizar Habash, Tuleen A AbuSamra, Marah Kaabneh, Rashed Al-Marmouri, Ghaid Abusharar, Rinal Khasawneh, Zaid Al-Tal, Mihar Raouf, Qusai T Ashourah, Shehab Aldeen Allahawyah, Aseel Aldabbas

**Affiliations:** 1 Pediatrics, Jordanian Ministry of Health, Zarqa, JOR; 2 Faculty of Medicine, University of Jordan, Amman, JOR; 3 Internal Medicine, Jordanian Royal Medical Services, Amman, JOR; 4 Internal Medicine, King Abdullah University Hospital, Irbid, JOR; 5 Medicine, University of Jordan, Amman, JOR; 6 General Practice, Jordanian Royal Medical Services, Amman, JOR; 7 General Practice, Jordan University Hospital, Amman, JOR; 8 Faculty of Medicine, Jordan University of Science and Technology, Irbid, JOR; 9 Faculty of Medicine, Hashemite University, Zarqa, JOR; 10 Family Medicine, Jordan University Hospital, Amman, JOR; 11 Internal Medicine, Al Nadeem Hospital, Madaba, JOR; 12 Faculty of Medicine, Al-Balqa’ Applied University, As-Salt, JOR

**Keywords:** gut inflammation, helicobacter pylori, host microbe interaction, immune modulation, inflammatory bowel disease, irritable bowel syndrome

## Abstract

*Helicobacter pylori* (*H. pylori*) infection has been extensively studied for its potential association with irritable bowel syndrome (IBS), with prior meta-analyses reporting inconsistent findings. This study aimed to perform a systematic review and meta-analysis of observational studies restricted to those providing reconstructable 2×2 contingency data, thereby enhancing transparency and reproducibility. A systematic search of PubMed, EMBASE, and the Cochrane Central Register of Controlled Trials (CENTRAL) was conducted from database inception to present. Eligible studies were observational (cross-sectional or case-control), included adult populations with appropriate control groups, and reported extractable 2×2 data for *H. pylori* infection status. Pooled odds ratios (ORs) with 95% confidence intervals (CIs) were calculated using a random-effects model. Four studies met the inclusion criteria for IBS. The pooled analysis demonstrated no statistically significant association between *H. pylori* infection and IBS (OR 1.20; 95% CI 0.63-2.29; p = 0.57; I² = 63.4%). A single case-control study evaluating inflammatory bowel disease (IBD) demonstrated an inverse association with ulcerative colitis, which is presented narratively due to insufficient studies for meta-analysis. In this restricted analysis, no significant association between *H. pylori* infection and IBS was observed. These findings should be interpreted cautiously, given the limited number of studies and reliance on unadjusted estimates.

## Introduction and background

*Helicobacter pylori* is a Gram-negative, microaerophilic pathogen capable of establishing persistent gastric colonization, thereby triggering sustained mucosal inflammatory responses through coordinated activation of both innate and adaptive immune effector mechanisms [1]. This chronic infection has been definitively linked to the pathogenesis of multiple gastroduodenal conditions, including chronic active gastritis, peptic ulcer disease, gastric adenocarcinoma, and mucosa-associated lymphoid tissue lymphoma [2].

Irritable bowel syndrome (IBS) constitutes a highly prevalent functional gastrointestinal disorder characterized by recurrent abdominal pain in association with altered defecatory patterns, occurring in the absence of demonstrable structural abnormalities on conventional diagnostic evaluation [3]. Although historically categorized as purely functional, accumulating evidence implicates low-grade mucosal inflammation, enhanced intestinal permeability, immune cell activation, and dysregulation of the gut-brain bidirectional communication axis in its underlying pathophysiology.

In contradistinction, inflammatory bowel disease (IBD) encompasses chronic immune-mediated conditions, principally Crohn’s disease and ulcerative colitis, characterized by persistent, destructive intestinal inflammation with consequent disruption of epithelial barrier integrity [4]. Considerable epidemiological effort has been devoted to investigating whether *H. pylori* colonization influences susceptibility to either IBS or IBD. While certain investigations report increased prevalence of IBS symptoms among *H. pylori*-positive individuals, others demonstrate no significant association [5]. Conversely, multiple observational studies and meta-analyses have suggested an inverse relationship between *H. pylori* infection and IBD, potentially mediated through immunoregulatory effects on T-helper cell differentiation pathways [6].

Previous meta-analyses have reported positive associations between *H. pylori* infection and IBS. A 2023 analysis including 27 studies reported a pooled OR of 1.68 [7], while others have demonstrated similar findings [5,6]. A 2026 meta-analysis of IBD, including 44 studies, reported a pooled OR of 0.43 [8]. The present study applies restrictive inclusion criteria limited to studies providing reconstructable 2×2 contingency data. This approach is designed to ensure greater data transparency and reproducibility. The present study does not aim to replace existing meta-analyses but rather to provide a complementary analysis using restricted inclusion criteria. Additionally, recent Mendelian randomization studies have suggested no causal relationship between *H. pylori* infection and IBS using genetic variants [9].

## Review

Materials and methods

Literature Search Strategy

A comprehensive search was performed in PubMed, EMBASE, and the Cochrane Central Register of Controlled Trials (CENTRAL) from database inception to December 2025. The search strategy integrated Medical Subject Headings (MeSH) and free-text terms associated with "Helicobacter pylori", "H. pylori", "irritable bowel syndrome", "IBS", "inflammatory bowel disease", "ulcerative colitis", and "Crohn’s disease", utilizing Boolean operators (AND/OR). The reference lists of eligible articles were manually reviewed to identify additional relevant studies. The structured database search framework is detailed in Table 1. This systematic review and meta-analysis was carried out in accordance with the Preferred Reporting Items for Systematic Reviews and Meta-Analyses (PRISMA) 2020 guidelines [10].

**Table 1:** Database search strategy for the systematic review of Helicobacter pylori and gastrointestinal disorders

Step	Search Query
1	"Helicobacter pylori"[MeSH] OR "H. pylori"
2	"irritable bowel syndrome"[MeSH] OR IBS
3	"inflammatory bowel disease"[MeSH] OR ulcerative colitis OR Crohn's disease
4	1 AND 2
5	1 AND 3
6	4 OR 5

Eligibility Criteria

Eligible investigations employed observational designs (cross-sectional or case-control), enrolled adult populations, incorporated appropriate control groups, and provided complete 2×2 contingency data enabling reconstruction of *H. pylori* infection status among both case and control participants. Studies were excluded if they involved pediatric populations, comprised case reports, case series, editorials, or systematic reviews, lacked control groups, evaluated only post-eradication status, utilized non-human subjects, or failed to provide reconstructable quantitative data. No eligible cohort studies meeting the inclusion criteria were identified.

Study Selection and Data Extraction

**Figure 1:**
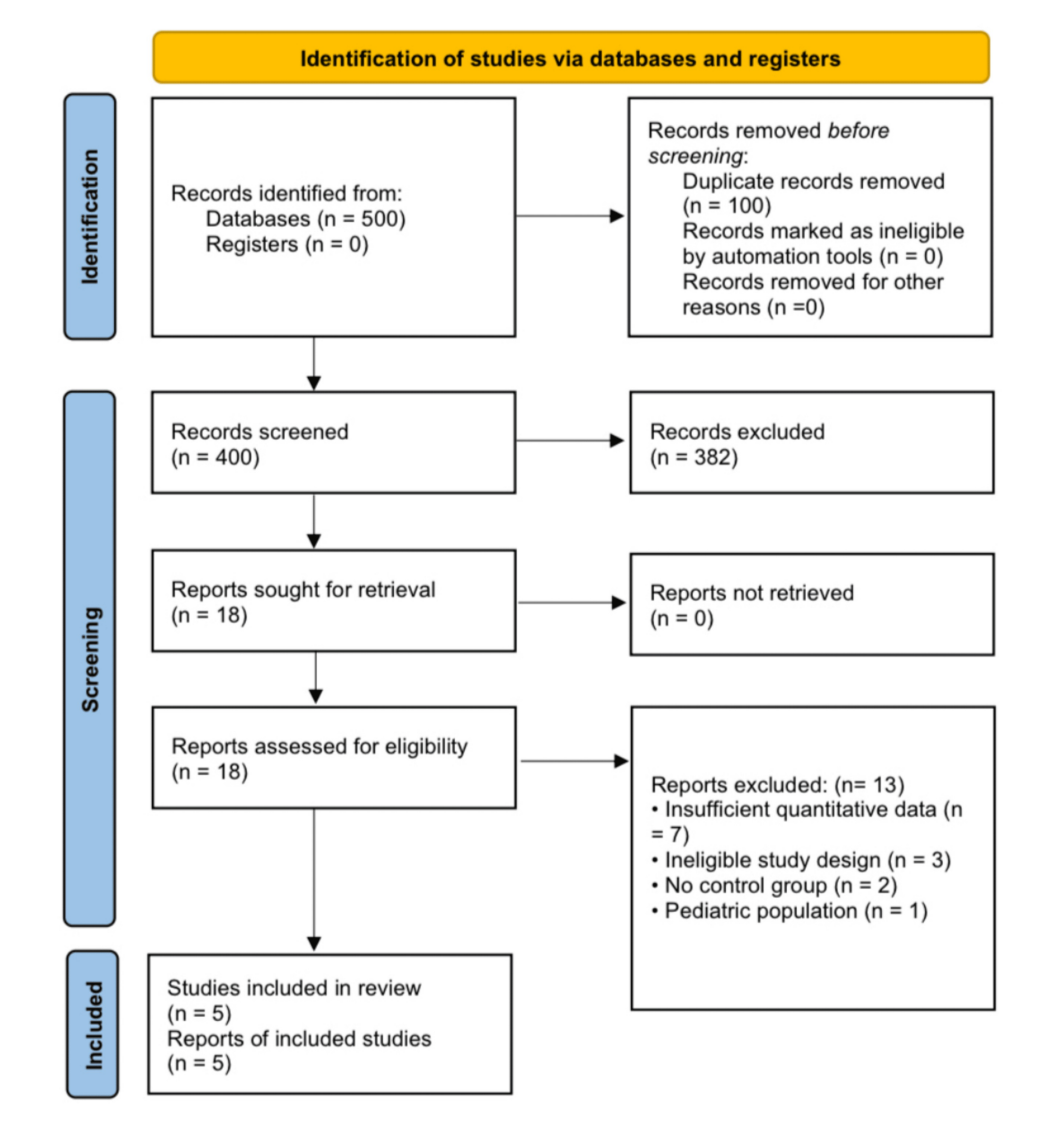
PRISMA flow diagram of study selection PRISMA: Preferred Reporting Items for Systematic Reviews and Meta-Analyses

The preliminary search identified 500 records (100%). A total of 400 (80%) remained for title and abstract screening after excluding 100 duplicate records (20%). Of these, 382 records (95.5%) were excluded based on pre-established criteria. Eighteen full-text articles were assessed for eligibility, of which 13 were excluded (seven due to insufficient quantitative data, three due to ineligible study design, two due to the absence of control groups, and one due to the inclusion of a pediatric population). Ultimately, five observational studies (four evaluating IBS and one evaluating IBD) were included in the quantitative synthesis [11-15]. The PRISMA flow diagram illustrating the study selection process is presented in Figure 1.

All studies were independently screened by two reviewers, who also collected the relevant data, including author, year, country, study design, sample size, *H. pylori* detection method, and contingency data for effect size reconstruction.

Quality Assessment

The methodological quality of the included studies was assessed using the Newcastle-Ottawa Scale (NOS), a validated tool for evaluating the quality of non-randomized studies in meta-analyses [16]. The NOS assesses studies across three domains: selection of study groups, comparability of groups, and ascertainment of exposure or outcome. Studies scoring ≥7 stars were classified as high quality, scores of 5-6 as moderate quality, and scores <5 as low quality. Among the selected studies, four were rated as high quality and one as moderate quality, with none classified as low quality [11-15].

Statistical Analysis

Odds ratios (ORs) with 95% confidence intervals were derived from reconstructed 2×2 contingency tables for every study. To address potential variability among studies, pooled effect estimates were calculated using the DerSimonian-Laird random-effects approach. Cochran’s Q test was utilized to evaluate statistical heterogeneity, which was further measured with the I² statistic. Given the small number of studies included, formal assessments for publication bias were not performed. Statistical significance was established as a two-sided p-value of less than 0.05. All meta-analyses were conducted using Review Manager (RevMan) version 5.4 (The Cochrane Collaboration, London, UK). All pooled estimates are based on unadjusted ORs derived from 2×2 contingency data, as adjusted estimates were not uniformly available across studies.

Results

Study Characteristics

The five included investigations comprised four evaluating IBS and one examining IBD [11-15]. Studies originated from European, Asian, and Middle Eastern populations and employed diverse diagnostic modalities, including serological testing, urea breath testing, histopathological assessment, and stool antigen testing (Table 2).

**Table 2:** Study characteristics and H. pylori detection methods of included studies NOS: Newcastle-Ottawa Scale

Study (year)	Country	Design	Condition	Cases/Controls (N)	*H. pylori* detection method	NOS score
Agreus L et al. (1995) [11]	Sweden	Cross-sectional	IBS	50/50	Serology	7
Su YC et al. (2000) [12]	Taiwan	Case-control	IBS	69/52	Serology	7
Gerards C et al. (2001) [13]	Germany	Case-control	IBS	31/15	Urea breath test	6
Yakoob J et al. (2012) [14]	Pakistan	Case-control	IBS	170/160	Histology	7
Ali I et al. (2022) [15]	Palestine	Case-control	IBD	35/105	*H. pylori* stool antigen test	6

To ensure transparency and facilitate reproducibility of pooled estimates, reconstructed 2×2 contingency data for *H. pylori* infection status among case and control participants were compiled from all included studies (Table 3). The table presents absolute frequencies with corresponding percentages for *H. pylori*-positive and *H. pylori*-negative participants stratified by disease type and group status.

**Table 3:** 2×2 Contingency data for H. pylori positive and negative results in IBS and IBD cases compared to controls IBS: irritable bowel syndrome; IBD: inflammatory bowel disease

Study (year)	Disease type	Group	*H. pylori* positive (N, %)	*H. pylori* negative (N, %)	Total (N)
Agréus et al. (1995) [11]	IBS	Cases	12 (24.0%)	38 (76.0%)	50
Agréus et al. (1995) [11]	IBS	Controls	20 (40.0%)	30 (60.0%)	50
Su et al. (2000) [12]	IBS	Cases	38 (55.1%)	31 (44.9%)	69
Su et al. (2000) [12]	IBS	Controls	18 (34.6%)	34 (65.4%)	52
Gerards et al. (2001) [13]	IBS	Cases	8 (25.8%)	23 (74.2%)	31
Gerards et al. (2001) [13]	IBS	Controls	4 (26.7%)	11 (73.3%)	15
Yakoob et al. (2012) [14]	IBS	Cases	94 (55.3%)	76 (44.7%)	170
Yakoob et al. (2012) [14]	IBS	Controls	72 (45.0%)	88 (55.0%)	160
Ali et al. (2022) [15]	IBD	Cases	5 (14.3%)	30 (85.7%)	35
Ali et al. (2022) [15]	IBD	Controls	44 (41.9%)	61 (58.1%)	105

Quantitative Synthesis

**Figure 2:**
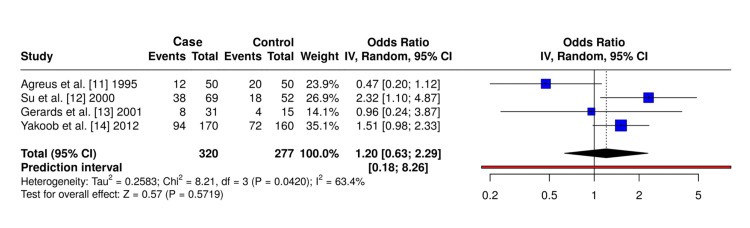
Forest plot of pooled odds ratios (ORs) using an inverse variance random-effects model for the association between Helicobacter pylori infection and irritable bowel syndrome (IBS)

Association with IBS: Pooled analysis demonstrated no statistically significant association between *H. pylori* infection and IBS (OR 1.20; 95% CI 0.63-2.29; p = 0.57), with moderate heterogeneity observed (I² = 63.4%) across studies [11-14]. The corresponding forest plot is shown in Figure 2.

Association with IBD: Meta-analysis was not performed for IBD because only one eligible study met the inclusion criteria. The single case-control study examining IBD demonstrated a significant inverse association between *H. pylori* infection and ulcerative colitis (OR 0.23; 95% CI 0.083-0.643; p = 0.003) [15]. In this investigation, *H. pylori* prevalence was 14.3% among IBD cases compared with 41.9% among control participants. These findings are presented narratively.

Discussion

This systematic review found no statistically significant association between *H. pylori* infection and IBS (OR 1.20; 95% CI 0.63-2.29) when analysis was restricted to studies providing reconstructable 2×2 data. Moderate heterogeneity was observed (I² = 63.4%). This null finding differs from previously published larger meta-analyses that reported positive associations ranging from OR 1.68 to 1.85 [5-7].

The moderate heterogeneity observed among IBS studies likely reflects multiple contributing factors, including geographic population differences, variable baseline *H. pylori* prevalence, diverse diagnostic modality utilization, and heterogeneous IBS diagnostic criteria application. Serological testing may indicate prior exposure rather than active infection, potentially introducing misclassification bias compared with urea breath testing or histopathological confirmation. Relative to prior meta-analyses [5-7], the present review restricted inclusion to investigations providing reconstructable 2×2 contingency data, thereby enhancing statistical transparency and reproducibility. Subgroup analyses stratified by geography or study design were not feasible due to the small number of included studies.

A methodological consideration in this field is whether *H. pylori* is associated with IBS itself or with overlapping symptoms from functional dyspepsia. *H. pylori* is a known cause of functional dyspepsia. The discrepancy between the present null findings and previous positive meta-analyses may be partially explained by symptom overlap between IBS and *H. pylori*-associated functional dyspepsia. Without individual patient data, it is difficult to determine whether prior positive associations were driven by undiagnosed dyspepsia cases. This distinction is important for clinical interpretation, as *H. pylori* eradication may improve dyspeptic symptoms but would not be expected to alter pure IBS pathophysiology. Clinically, these findings suggest that routine *H. pylori* testing and eradication are unlikely to benefit IBS patients without dyspeptic symptoms.

Previous meta-analyses have pooled larger numbers of studies and often incorporated adjusted estimates. The present analysis takes a deliberately restricted approach by limiting inclusion to studies with reconstructable 2×2 contingency data, prioritizing data transparency and reproducibility. The discrepancy between the present null finding and previously reported positive associations may reflect differences in inclusion criteria, confounding, or publication bias. The present study should not be interpreted as a replacement for existing meta-analyses but rather as a complementary analysis using restricted inclusion criteria. The 2023 IBS meta-analysis specifically reported subgroup findings based on study design, IBS criteria, and the *H. pylori* testing method, indicating that the field is heterogeneous and method-sensitive. Recent Mendelian randomization studies have suggested no causal relationship between *H. pylori* infection and IBS using genetic variants [9]. This genetic evidence is not subject to confounding or reverse causation and is broadly consistent with the present findings.

Turning to IBD, although IBS and IBD are distinct conditions, the relationship between *H. pylori* and IBD warrants brief mention. The single eligible IBD study demonstrated an inverse association between *H. pylori* infection and ulcerative colitis (OR 0.23) [15]. This finding aligns with a larger 2026 meta-analysis of 44 studies that reported a significant negative association between *H. pylori* colonization and IBD (OR 0.43; 95% CI 0.35-0.52) [8]. However, because only one IBD study met the criteria for this review, the IBD evidence should be interpreted as narrative support rather than pooled meta-analytic evidence. Observed geographic variation in prior work suggests that gene-environment interactions may modify this relationship [17].

Several limitations warrant acknowledgment. The use of unadjusted ORs derived from 2×2 contingency data means that confounding bias was not controlled, which is a limitation given the observational nature of the included studies. Previous meta-analyses that pooled adjusted ORs may therefore provide more confounder-resistant estimates. The small number of studies (n = 4 for IBS) limited statistical power and precision. No eligible cohort studies were identified. Moderate heterogeneity (I² = 63.4%) could not be fully explored due to the limited number of studies. The IBD evidence is based on a single study and is not intended to replace the existing 2026 meta-analysis of 44 studies [8]. Additionally, even high-quality observational studies cannot eliminate unmeasured confounding. More broadly, the restricted inclusion criteria used in this review may have introduced selection bias by excluding larger studies that did not report extractable contingency tables.

## Conclusions

In this systematic review and meta-analysis restricted to studies with reconstructable 2×2 data, no statistically significant association was observed between *H. pylori* infection and IBS. These findings differ from prior meta-analyses but are consistent with recent evidence suggesting no causal relationship. Strengths of this review include adherence to PRISMA guidelines, the use of restrictive inclusion criteria to enhance data transparency, formal quality assessment using the NOS, and contextualization of findings within the existing literature. However, several limitations should be acknowledged, including reliance on unadjusted ORs, inability to control for confounding, small number of included studies, absence of eligible cohort designs, and moderate heterogeneity across studies. The present analysis should be interpreted as a complementary, methodologically restricted evaluation rather than a replacement for larger syntheses. Accordingly, these findings should be considered hypothesis-generating rather than definitive. Future research should prioritize large-scale prospective cohort studies with standardized diagnostic criteria and transparent reporting of both crude and adjusted estimates.
